# *De Novo* Genome Assembly of Grapevine Yellow Speckle Viroid 1 from a Grapevine Transcriptome

**DOI:** 10.1128/genomeA.00496-15

**Published:** 2015-05-21

**Authors:** Yeonhwa Jo, Hoseong Choi, Ju-Yeon Yoon, Seung-Kook Choi, Won Kyong Cho

**Affiliations:** a Department of Agricultural Biotechnology, College of Agriculture and Life Sciences, Seoul National University, Seoul, Republic of Korea; b Department of Horticultural Environment, Virology Unit, National Institute of Horticultural and Herbal Science, RDA, Wan-Ju, Republic of Korea

## Abstract

Grapevine yellow speckle viroid 1 (GYSVd1), which is a member of the genus *Apscaviroid*, causes yellow speckle disease in grapevines. Here, we report the complete *de novo* genome assembly of GYSVd1 from the grapevine transcriptome and identified 10 single nucleotide polymorphisms of GYSVd1 across the grapevine populations.

## GENOME ANNOUNCEMENT

Viroids are the smallest plant pathogens known and are composed of a circular single-stranded RNA genome that does not encode any proteins (1). The replication and movement of viroids are highly dependent on the infected host expression machinery. Thus far, two different viroid families, *Pospiviroidae* and *Avsunviroidae*, which are replicated in the nucleus and chloroplast, respectively, have been identified (1). Grapevines serve as the host for several viroids, including hop stunt viroid (HSVd), grapevine yellow speckle viroid 1 (GYSVd1), grapevine yellow speckle viroid 2 (GYSVd2), Australian grapevine viroid (AGVd), and *Citrus exocortis* viroid (CEVd) (2, 3). Of these viroids, GYSVd1 and GYSVd2, members of the genus *Apscaviroid*, induce yellow speckle disease symptoms, which are strongly affected by environmental conditions (4). GYSVd1 is generally mechanically transmitted by grafting.

During our screening process to identify viroids infecting grapevines, we found that the grapevine transcriptome was infected by GYSVd1. The transcriptome was prepared from the grape berries without seeds from the 10-year-old grapevine cultivar Cabernet Sauvignon, grown in China (5). The library was sequenced using a single-end Illumina HiSeq 2000 platform. We performed *de novo* transcriptome assembly using the Trinity program (version 2.0.2) (6). The obtained contigs were blasted against viroid reference sequences. Two assembled contigs, 214 and 204 nucleotides (nt) long, were highly matched to the known GYSVd1 reference genome sequence (accession no. NC_001920.1). After alignment of the two contigs on the reference GYSVd1 genome, we obtained a complete GYSVd1 genome sequence with a length of 368 nt. We named the newly identified GYSVd1 isolate Cabernet Sauvignon (CS) (accession no. KP993474). The GYSVd1 isolate CS was closest to the GYSVd1 (accession no. EU682454.1) isolated from the cultivar Nebbiolo in Italy, with 99% sequence identity (359/364) (7). Viroids are highly mutated and present as quasispecies, including several variants. We therefore examined single nucleotide variations (SNVs) of GYSVd1 in the grapevine cultivar CS by aligning all raw data against the genome of the GYSVd1 isolate CS using the BWA program (8). To identify SNVs, variant calling was performed by SAMtools (9). We found 10 SNVs at nucleotide positions 59, 84, 85, 135, 141, 145, 305, 307, 309, and 317, indicating the presence of quasispecies of GYSVd1 in the single grapevine cultivar. In particular, the nucleotide at position 145 exhibited indels (insertions and deletions) from CT to C. Taken together, our study identifies the complete genome of GYSVd1 by *de novo* assembly using a grapevine transcriptome. In addition, the SNVs of GYSVd1 demonstrate the presence of several variants of GYSVd1 across the same grapevine vineyard.

### Nucleotide sequence accession number. 

The genome sequence of grapevine yellow speckle viroid 1 isolate Cabernet Sauvignon has been submitted to GenBank (accession no. KP993474).
